# Social Media and Selfie-Related Mortality Amid COVID-19: Interrupted Time Series Analysis

**DOI:** 10.2196/42857

**Published:** 2023-09-25

**Authors:** Sarit Kang-Auger, Antoine Lewin, Aimina Ayoub, Marianne Bilodeau-Bertrand, Sophie Marcoux, Nathalie Auger

**Affiliations:** 1 Faculty of Medicine University of Montreal Montreal, QC Canada; 2 Department of Obstetrics and Gynecology University of Sherbrooke Sherbrooke, QC Canada; 3 Medical Affairs and Innovation Héma-Québec Saint-Laurent, QC Canada; 4 University of Montreal Hospital Research Centre Montreal, QC Canada; 5 Institut national de santé publique du Québec Montreal, QC Canada; 6 School of Public Health University of Montreal Montreal, QC Canada; 7 Department of Epidemiology, Biostatistics, and Occupational Health McGill University Montreal, QC Canada

**Keywords:** COVID-19, injury, mortality, mortality rate, web-based news, selfie, social media, time series regression

## Abstract

**Background:**

COVID-19 had a considerable impact on mortality, but its effect on behaviors associated with social media remains unclear. As travel decreased due to lockdowns during the pandemic, selfie-related mortality may have decreased, as fewer individuals were taking smartphone photographs in risky locations.

**Objective:**

In this study, we examined the effect of the COVID-19 pandemic on trends in selfie-related mortality.

**Methods:**

We identified fatal selfie-related injuries reported in web-based news reports worldwide between March 2014 and April 2021, including the deaths of individuals attempting a selfie photograph or anyone else present during the incident. The main outcome measure was the total number of selfie-related deaths per month. We used interrupted time series regression to estimate the monthly change in the number of selfie-related deaths over time, comparing the period before the pandemic (March 2014 to February 2020) with the period during the pandemic (March 2020 to April 2021).

**Results:**

The study included a total of 332 selfie-related deaths occurring between March 2014 and April 2021, with 18 (5.4%) deaths during the pandemic. Most selfie-related deaths occurred in India (n=153, 46.1%) and involved men (n=221, 66.6%) and young individuals (n=296, 89.2%). During the pandemic, two-thirds of selfie-related deaths were due to falls, whereas a greater proportion of selfie-related deaths before the pandemic were due to drowning. Based on interrupted time series regression, there was an average of 1.3 selfie-related deaths per month during the pandemic, compared with 4.3 deaths per month before the pandemic. The number of selfie-related deaths decreased by 2.6 in the first month of the pandemic alone and continued to decrease thereafter.

**Conclusions:**

Our findings indicate that the COVID-19 pandemic led to a marked decrease in selfie-related mortality, suggesting that lockdowns and travel restrictions likely prevented hazardous selfie-taking. The decrease in selfie-related mortality occurred despite a potential increase in social media use during the pandemic.

## Introduction

The COVID-19 pandemic was linked to considerable morbidity and mortality [1,2], but its impact on mortality associated with social media behavior remains unknown. In 2014, reports of selfie-related injuries involving young people and tourists taking sensational photographs for social media started appearing in international news [3]. Afterward, selfie-related incidents continued to increase, eventually becoming a prominent complication of extreme social media behavior, where individuals sought to post photos of themselves in risky locations [3]. Selfie-related mortality drew significant attention in the media, with selfie-related deaths continuing to increase around the world [3-5]. Although it is evident that selfie-related mortality was on the rise before the onset of COVID-19, the impact of the pandemic on these trends over time remains uncertain.

Lockdown measures and travel restrictions during the pandemic had the potential to reduce the number of selfie-related deaths. In an effort to minimize the spread of COVID-19, many countries imposed social distancing measures, stay-at-home orders, curfews, restrictions on public gatherings, travel bans, as well as school and business closures, aimed at reducing human mobility and interaction [6,7]. These measures helped reduce COVID-19 mortality but also had an unintended effect on mortality from different types of injury [8-10]. Mortality from accidental drug overdose appeared to increase [8], while mortality due to unintentional injuries associated with travel, such as motor vehicle accidents, decreased at the start of the pandemic [9,10]. As selfie-related mortality is also considered an unintentional injury potentially affected by travel restrictions during lockdowns, a reduction in this specific cause of death is possible. In this study, we examined the impact of the pandemic on selfie-related mortality trends around the world.

## Methods

### Procedure

We used a quasi-experimental design to assess temporal trends in the monthly number of selfie-related deaths during the pandemic. We analyzed two time periods: the period before the pandemic, which began in March 2014 and ended in February 2020, and the first year of the pandemic, which was from March 2020 until April 2021. The World Health Organization declared COVID-19 a pandemic in March 2020 [11]. Most countries implemented lockdowns in March and restricted travel during the first year of COVID-19.

Through a Google search for the terms “selfie” and “death” or “mortality,” we identified all selfie-related deaths that were documented in web-based English media reports around the world. We used the Wikipedia selfie-related death registry to identify any missed reports [12].

We defined a selfie-related death as the unintentional death of a person attempting a selfie photograph with an electronic device or the death of any other individual involved in the incident. We excluded nonfatal selfie injuries and injuries missing the month of death. We documented the date, country, and cause of death, along with the sex and age of the victim.

### Statistical Analysis

We used interrupted time series regression to plot the monthly change in the number of selfie-related deaths before and during the pandemic [13]. Using autoregressive models with a multiplicative interaction term involving time and the start of the pandemic, we estimated trends in the monthly number of selfie-related deaths before the pandemic, in March 2020, and the subsequent duration of the pandemic [13]. Autoregressive models allow for the control of seasonality and correlation in the number of deaths between months [13]. Interrupted time series regression is ideal for isolating the impact of sudden unplanned events, such as the pandemic, that started at a specific point in time [14]. We analyzed the data using SAS v9.4 (SAS Institute Inc). We have included the data and syntax as Multimedia Appendix 1 and 2.

### Ethical Considerations

As media reports were publicly available, we did not seek additional ethics review.

## Results

We identified 332 selfie-related deaths between March 2014 and April 2021, including 18 (5.4%) deaths during the pandemic. Most victims were men (n=221, 66.6%) and younger than 40 years (n=296, 89.2%). Countries with the highest number of reported selfie-related deaths included India (n=153, 46.1%), the United States (n=28, 8.4%), and Russia (n=20, 6.0%). The distribution of age, sex, and the country of selfie-related deaths remained similar both before and during the pandemic.

Overall, the most common causes of death were drowning (n=118, 35.5%), falls (n=101, 30.4%), and transport-related injuries (n=68, 20.5%). Falls were more prominent among deaths during the pandemic (11/18, 61.1%). Before the pandemic, both falls (90/314, 28.7%) and drowning (115/314, 36.6%) contributed to selfie-related mortality.

**Figure 1:**
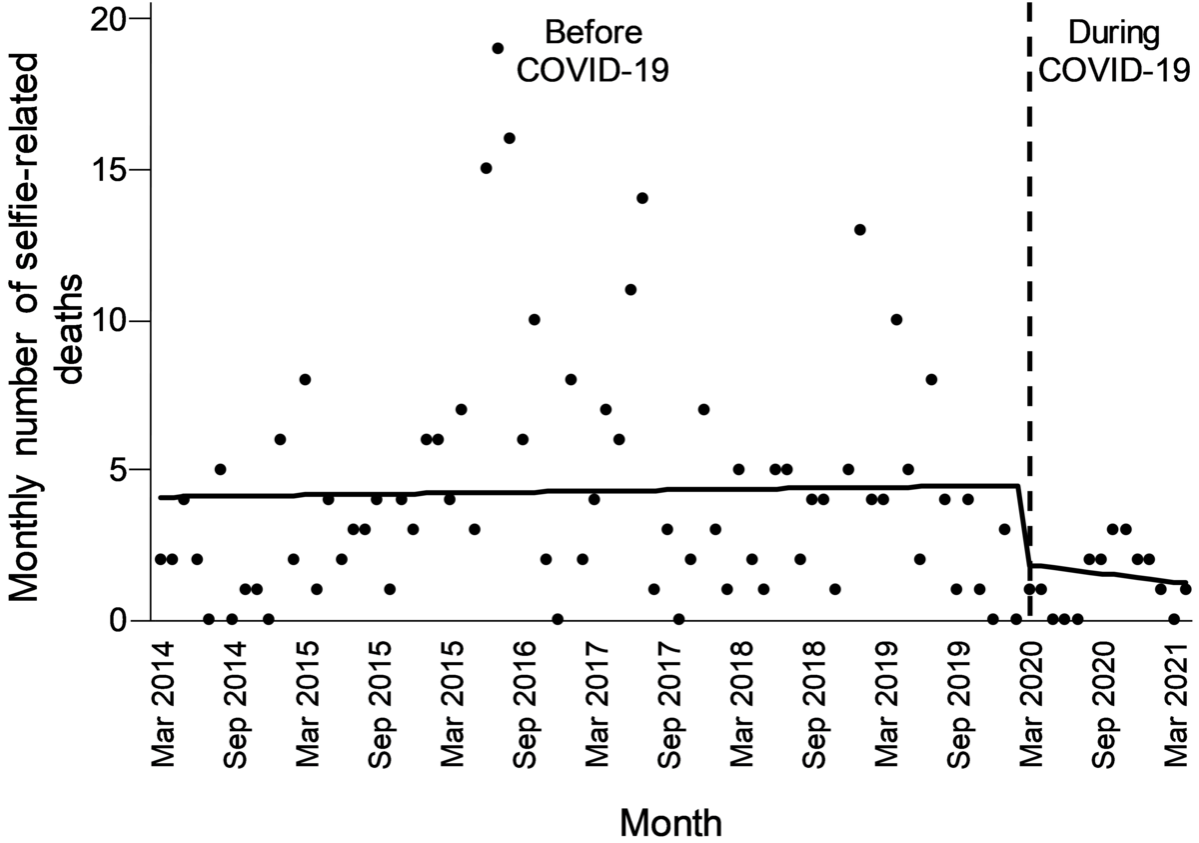
Monthly number of selfie-related deaths before and during the COVID-19 pandemic (March 2014 to April 2021). Dots represent the number of deaths per month. The solid line represents the predicted trend in the monthly number of deaths from the interrupted time series regression model.

The monthly number of selfie-related deaths increased steadily between March 2014 and February 2020 but decreased sharply at the start of the pandemic (Figure 1). Before the pandemic, the average number of selfie-related deaths per month was 4.3, with a monthly increase of 0.006 deaths. In contrast, there were 1.3 selfie-related deaths per month during the pandemic. The onset of lockdowns led to a decrease of 2.6 deaths in March 2020 alone. Throughout the remaining duration of the pandemic, the number of selfie-related deaths decreased by 0.05 each month; however, this trend was not statistically significant, due to the low number of deaths within the shorter follow-up period.

## Discussion

### Principal Findings

The findings of this study suggest that COVID-19 led to a reduction in selfie-related mortality around the world, adding to the growing literature on some of the unexpected effects of the pandemic on unintentional injuries [8-10]. Lockdown measures and travel restrictions appear to have limited the number of opportunities for taking risky selfies during the pandemic, consequently reducing the number of deaths attributed to this cause.

Selfies taken in dangerous circumstances often draw considerable attention on social media. Overconfidence, peer pressure, attention seeking, and competition are thought to drive the desire to post hazardous selfies [15]. Researchers are increasingly pointing out the importance of studying this behavior [3,15]. Existing studies of media reports have shown that selfie-related deaths are particularly common among men, younger individuals, and in India [3-5]. These same factors contributed to selfie-related mortality during the pandemic in our study, although the number of deaths was much lower.

The decrease in selfie-related mortality occurred despite the increased use of social media during the pandemic, suggesting that selfie-related behaviors may have been replaced by other web-based activities [16]. Social media was a primary source of information during the pandemic [16]. Social media also became the main tool for social relations. A survey of youth from Belgium indicated that adolescents frequently used social media to reduce loneliness during lockdowns [17]. Platforms like TikTok, known for spreading dangerous challenges, became very popular [18,19]. In one survey, users of dating apps, such as Tinder, were more likely to have risky drinking behaviors during the pandemic [20]. Therefore, selfie-taking may have been replaced by other risky behaviors on social media platforms that boomed during the pandemic.

Other types of unintentional mortality were also affected by lockdowns, especially motor vehicle accidents [9,10]. Studies from the United States and Peru have shown that motor vehicle fatalities decreased in the first months of the pandemic [9,10]. Selfie-related mortality shares common risk factors with motor vehicle accidents, such as young age, male sex, substance use, and reckless behavior [3,21,22]. This suggests that lockdowns may have had beneficial effects for individuals prone to impulsivity. However, other transport-related accidents, such as cycling injuries, increased during the first year of the pandemic [23].

Prevention of selfie-related mortality has proven challenging, as no-selfie zones and physical barriers have not been successful in reducing deaths. Novel strategies focused on travel or tourism may be helpful, as lockdowns and mobility restrictions were associated with a considerable decrease in selfie-related mortality in this study. Regulating the use of psychoactive substances in risky areas and spreading awareness on social media may be additional tools to prevent selfie-related mortality.

### Limitations

We based our analysis on published news articles and may have underestimated the total burden of selfie-related mortality, particularly if deaths were not covered by reporters or were covered in languages other than English. Deaths could also be underreported because some news reports may have failed to identify selfies as the underlying cause of death. However, there is no reason to suspect that misclassification changed over time. We could not calculate incidence rates for selfie-related mortality, as the total number of individuals taking selfies was not available. We analyzed the monthly number of selfie-related deaths, but we could not account for unknown population decreases in countries, which could have introduced bias into the results. Due to the low number of deaths during the pandemic, we were unable to stratify the interrupted time series by age, sex, nationality, or place of death.

### Conclusions

This study suggests that selfie-related mortality decreased during the first year of the pandemic, owing to lockdowns and travel restrictions that limited the number of selfies taken in risky locations. The findings of this study can help inform strategies to prevent selfie-related mortality in the postpandemic period. Prevention strategies could focus on limiting travel or access to dangerous locations, raising awareness through social media about the risks associated with selfies, and encouraging other forms of safe social media behavior.

## Appendix

Multimedia Appendix 1 Data used for interrupted time series regression of selfie-related deaths during the COVID-19 pandemic.

Multimedia Appendix 2 Analytic syntax used for interrupted time series regression of selfie-related deaths during the COVID-19 pandemic.

## Data Availability

The data set generated and analyzed during this study is available in Multimedia Appendix 1.
