# Supplementary material for: Social Media and Selfie-Related Mortality Amid COVID-19: Interrupted Time Series Analysis
Source: JMIR Form Res. 2023 Sep 25;7:e42857. doi: 10.2196/42857 (PMC10521909; doi:10.2196/42857)
Supplement: Multimedia Appendix 2 [file formative_v7i1e42857_app2.docx]

**Appendix 2** Analytic syntax used for interrupted time series regression of selfie deaths during Covid-19

*****************************************************************************

Objective: Data preparation and analysis for project on COVID-19 and selfie-related deaths

Author: Aimina Ayoub, Sarit Kang-Auger, and Marianne Bilodeau-Bertrand

Date: 11/11/2021

SAS version 9.4

*****************************************************************************

Table of contents

STEP 1: Import data from Excel

STEP 2: Data preparation

STEP 3: No. selfie deaths

STEP 4: Interrupted time series regression

a) Dataset

b) Analysis

c) Figure

*****************************************************************************;

LIBNAME selfie "D:\";

/*** STEP 1: Import data from Excel ***/

**PROC** **IMPORT** OUT= selfie.data_selfiedeath

DATAFILE= "D:\JMIR_KangAuger_Data.xlsx"

DBMS=xlsx REPLACE;

SHEET="Selfie deaths";

**RUN**;

/*** STEP 2: Data preparation ***/

**DATA** selfie.data_selfiedeath2; SET selfie.data_selfiedeath;

*Create variables;

**Death;

Death = **1**;

LABEL Death = "Death 1Yes 0No";

**Sasdate;

FORMAT sasdate yymmdd10.;

IF day ne **.** and month ne **.** and year ne **.** THEN DO;

sasdate = mdy(month,day,year);

END;

LABEL Sasdate = "Date of death";

**Sex;

IF Sex = "M" THEN Male=**1**;

ELSE IF Sex = "F" THEN Male=**0**;

ELSE Male=**2**;

LABEL Male = "Sex 0Female 1Male 2Unknown";

**Age;

age_c=**.**;

IF (Age ne **.** and Age<**40**) or Age_range in("13-19","14-15","15-16","16-20","17-20","17-25","18-30","20-39","21-28","25-30","30-39") THEN age_c=**1**;

IF Age>=**40** or Age_range="50-59" THEN age_c=**0**;

LABEL age_c = "Age 0>=40 1<40 years";

**Pandemic;

pandemic=**0**;

IF (month>=**3** and year>=**2020**) or year>=**2021** THEN pandemic=**1**;

LABEL pandemic = "0Before pandemic 1During pandemic";

/*Exclusion*/

IF month=**.** THEN DELETE;

**RUN**;

**DATA** selfie.data_selfiedeath3;

RETAIN Death Day Month Year Sasdate age_c Male Country Cause pandemic;

SET selfie.data_selfiedeath2;

KEEP Death Day Month Year Sasdate age_c Male Country Cause pandemic;

**RUN**;

/*** STEP 3: No. selfie deaths ***/

**PROC FREQ** DATA=selfie.data_selfiedeath3;

TABLES (age_c Male Country Cause)*pandemic/MISSING;

**RUN**;

/*** STEP 4: Interrupted time series regression ***/

*a) Dataset;

**PROC SORT** DATA=selfie.data_selfiedeath3;

BY sasdate;

**RUN**;

/*Number of deaths by month and year*/

**PROC TABULATE** DATA=selfie.data_selfiedeath3 OUT=monthlymbb;

CLASS month year death/MISSING;

TABLE month*year, death/PRINTMISS MISSTEXT='0' RTS=**50**;

**RUN**;

**PROC** **SORT** DATA=monthlymbb;

BY year month;

**RUN**;

**DATA** monthlymbb; SET monthlymbb;

/*N is the number of deaths by month. If there is no death in a month, put the value 0 instead of .*/

monthly_death=N;

IF N=**.** THEN monthly_death=**0**;

/*Start March 2014*/

IF month<**3** and year=**2014** THEN DELETE;

/*End April 2021*/

IF month>**4** and year=**2021** THEN DELETE;

/*When the pandemic (interruption) started*/

pandemic=**0**;

IF (month>=**3** and year>=**2020**) or year>=**2021** THEN pandemic=**1**;

KEEP monthly_death pandemic;

**RUN**;

/*Time for each death*/

**DATA** monthlymbb; SET monthlymbb;

time=_N_;

**RUN**;

/*Time for deaths after the interruption (pandemic)*/

**DATA** monthlymbb; SET monthlymbb;

BY pandemic;

IF FIRST.pandemic THEN time_post = **0**;

time_post + **1**;

IF pandemic=**0** THEN time_post = **0**;

**RUN**;

*b) Analysis;

/*Reference:

Penfold RB, Zhang F. Use of interrupted time series analysis in evaluating health care quality improvements. Acad Pediatr 2013;13(6 Suppl):S38–S44. doi:10.1016/j.acap.2013.08.002

https://www.academicpedsjnl.net/article/S1876-2859(13)00210-6/pdf*/

ODS GRAPHICS ON;

**PROC** **AUTOREG** DATA = monthlymbb;

MODEL monthly_death = time Pandemic time_post

/PLOTS=ALL METHOD=ML NLAG=**12** DWPROB BACKSTEP;

OUTPUT OUT=outputdata PM=trendhat LCLM=lclm UCLM=uclm P=yhat LCL=lcl UCL=ucl;

**RUN**;

ODS GRAPHICS OFF;

*c) Figure;

**PROC** **SGPLOT** DATA=outputdata;

/*observed values*/

SCATTER X=time Y=monthly_death / MARKERATTRS = (COLOR=BLACK)

LEGENDLABEL = "Observed values";

/*predicted values*/

SERIES X=time Y=yhat / LINEATTRS =(COLOR=BLACK PATTERN=LongDash)

LEGENDLABEL = "Predicted series values";

/*predicted mean*/

SERIES X=time Y=trendhat / LINEATTRS =(COLOR= BLACK PATTERN=SOLID)

LEGENDLABEL = "Predicted mean (trend)";

/*LCLM and UCLM Lower and upper confidence limits for structural predicted values*/

BAND X=time UPPER=uclm LOWER=lclm / FILLATTRS= (COLOR= MEGR TRANSPARENCY=**.6**)

LEGENDLABEL = "Band for predicted trend";

/*x-axis*/

XAXIS LABELATTRS=(SIZE=**14**) VALUEATTRS=(SIZE=**14**);

/*y-axis*/

LABEL monthly_death = "Number of deaths per month before and after COVID-19 pandemic"

time = "Month";

YAXIS MIN=**0** MAX=**20** LABELATTRS=(SIZE=**14**) VALUEATTRS=(SIZE=**14**);

/*Interruption line*/

REFLINE **73** / AXIS=X LINEATTRS =(PATTERN=DOT COLOR=BLACK);

/*Legend*/

KEYLEGEND / VALUEATTRS=(FAMILY=Arial SIZE=**12**);

**RUN**;

ODS GRAPHICS OFF;

ODS CSV FILE = "D:\its_month.CSV"; **PROC** **PRINT** DATA = outputdata; **RUN**;

ODS CSV CLOSE;
